# Stage-dependent EZH2 methylation correlates with immune polarization, metabolic suppression, and unfavorable outcomes in hepatocellular carcinoma

**DOI:** 10.7150/ijms.119496

**Published:** 2025-10-01

**Authors:** Yi-Chung Chien, Guo-Wei Wu, Jia-Yan Wu, Liang-Chih Liu, Yi-Hsien Hsieh, Yung-Luen Yu

**Affiliations:** 1 Graduate Institute of Biomedical Sciences, China Medical University, Taichung 40402, Taiwan.; 2 Institute of Translational Medicine and New Drug Development, China Medical University, Taichung 40402, Taiwan.; 3 Center for Molecular Medicine, China Medical University Hospital, Taichung 40402, Taiwan.; 4 School of Medicine, College of Medicine, China Medical University, Taichung 40402, Taiwan.; 5 Department of Surgery, China Medical University Hospital, Taichung 40402, Taiwan.; 6 Institute of Medicine, Chung Shan Medical University, Taichung 40201, Taiwan.; 7 Department of Medical Research, Chung Shan Medical University Hospital, Taichung 40201, Taiwan.; 8 Cancer Biology and Precision Therapeutics Center, China Medical University, Taichung 40402, Taiwan.; 9 Office of Research and Development, Asia University, Taichung 41354, Taiwan.

**Keywords:** hepatocellular carcinoma, EZH2, methylation, immune polarization

## Abstract

Hepatocellular carcinoma (HCC), the most prevalent form of primary liver cancer, continues to pose significant clinical challenges globally. Enhancer of Zeste Homolog 2 (EZH2), a central component of the Polycomb Repressive Complex 2 (PRC2), possesses histone methyltransferase activity through its SET domain and is frequently overexpressed in various cancers. Nevertheless, the precise role and regulatory mechanisms of EZH2 in HCC remain inadequately defined. In this research, we evaluated the expression levels of EZH2 at the mRNA and protein stages in HCC samples and examined their correlation with clinical features and patient survival outcomes. Patients were categorized into early- and late-stage groups based on tumor grade. Our methylation analyses pinpointed two specific CpG sites within the EZH2 gene, cg08558971 and cg18416251, which exhibited inverse methylation patterns between tumor stages. One patient subgroup displayed high methylation at cg08558971 during early-stage disease and reduced methylation at cg18416251 during late-stage disease, while another subgroup demonstrated the reverse pattern. Further pathway enrichment analysis suggested these methylation variations might influence enhanced T-cell differentiation and suppress metabolic pathways. Additionally, correlation analyses consistently linked EZH2 expression to genes involved in these immune and metabolic pathways. Collectively, our data propose that EZH2 could serve as a meaningful independent prognostic biomarker for HCC, regulated by stage-dependent epigenetic changes that may drive tumor progression by modulating immune response and cellular metabolism.

## 1. Introduction

Hepatocellular carcinoma (HCC) is the predominant form of primary liver cancer, representing approximately 90% of cases globally 1. Despite recent advancements in diagnostic methods and therapeutic interventions, HCC continues to be one of the deadliest gastrointestinal malignancies and poses a significant public health issue worldwide 2, 3. Major risk factors for HCC include chronic infections with hepatitis B and C viruses, exposure to aflatoxins, and non-alcoholic steatohepatitis (NASH), frequently associated with metabolic syndrome and type 2 diabetes mellitus 1, 4, 5. Due to its often-asymptomatic progression, HCC is usually diagnosed at advanced stages, substantially limiting the efficacy of curative treatments and consequently resulting in poor patient prognosis 1, 6.

In early-stage HCC, surgical resection and liver transplantation are primary curative options, with thermal ablation serving as an effective alternative, especially suitable for smaller tumors or patients at higher surgical risk 1. For intermediate-stage disease, transarterial chemoembolization (TACE) remains the standard treatment approach. Recent developments in systemic therapies, such as multikinase inhibitors (e.g., sorafenib and lenvatinib) and immune checkpoint inhibitors (e.g., atezolizumab-bevacizumab and durvalumab-tremelimumab combinations), have significantly improved clinical outcomes in advanced HCC 7-9. Nonetheless, therapeutic responses vary widely, largely due to the molecular heterogeneity of HCC and the immunosuppressive characteristics of the tumor microenvironment (TME) 10-14.

Enhancer of Zeste Homolog 2 (EZH2) is a critical constituent of the polycomb repressive complex 2 (PRC2), catalyzing the trimethylation of histone H3 at lysine 27 to facilitate gene silencing 15, 16. Aberrant expression of EZH2 correlates with unfavorable prognosis in several cancers by promoting epithelial-mesenchymal transition (EMT), tumor progression, and metastasis 17-20. EZH2 exerts its oncogenic effects by repressing tumor suppressor genes such as p21, p16, and KLF2 through histone and DNA methylation and by modulating the activities of non-histone proteins like STAT3 and NF-κB, depending on cellular context 21-30. Additionally, EZH2 influences immune escape mechanisms by suppressing antigen presentation, boosting regulatory T-cell activity, and attracting immunosuppressive cells including myeloid-derived suppressor cells (MDSCs) and tumor-associated macrophages (TAMs) 31-33.

In this study, we aimed to investigate the clinical significance and epigenetic regulation of EZH2 in HCC. Analysis of clinical tumor samples demonstrated that elevated EZH2 expression correlated significantly with advanced disease stages and reduced patient survival. Importantly, methylation analysis identified two specific CpG sites within the EZH2 promoter, cg08558971 and cg18416251, showing distinct and inverse methylation patterns in early versus late stages of HCC. These sites appeared to be independently regulated during the disease progression. Gene co-expression analyses further indicated that genes associated with these methylation variations were significantly enriched in pathways related to Th1, Th2, and Th17 cell differentiation, whereas pathways associated with thermogenesis and peroxisomal metabolism were notably downregulated. These results collectively suggest that the epigenetic modulation of EZH2 may facilitate HCC progression by influencing critical immune and metabolic signaling pathways.

## 2. Materials and Methods

### 2.1 Patient cohorts

The Liver Hepatocellular Carcinoma (LIHC) dataset was obtained from The Cancer Genome Atlas (TCGA) data portal (https://gdc.cancer.gov). A total of 421 cases from the TCGA-LIHC cohort, each with available clinical data and mRNA expression profiles, were included for subsequent analyses 34.

### 2.2. EZH2 expression and overall survival analysis by UALCAN and The Human Protein Atlas (HPA)

The mRNA expression levels of EZH2 in solid normal tissues and hepatocellular carcinoma (HCC) samples from the TCGA-LIHC dataset were analyzed using the publicly available UALCAN web portal (https://ualcan.path.uab.edu/index.html) 35. Transcripts per million (TPM) values were computed using an in-house PERL (Practical Extraction and Report Language) script. TPM was selected as the metric for quantifying gene expression due to its improved accuracy in cross-sample comparisons relative to other commonly used measures such as FPKM (Fragments Per Kilobase of transcript per Million mapped reads) and RPKM (Reads Per Kilobase of transcript per Million mapped reads). The dataset provides gene-level expression estimates using log₂ (x + 1)-transformed RSEM-normalized counts 36. To evaluate the prognostic significance of EZH2 expression, HCC samples were stratified into two groups based on TPM values, high expression (TPM values above the upper quartile, n = 88) and low/medium expression (TPM values below the upper quartile, n = 277) 37 Kaplan-Meier survival analysis was subsequently performed to assess overall survival differences between these two groups. In addition, immunohistochemical (IHC) staining data for EZH2 in normal liver tissues and HCC tissues were obtained from The Human Protein Atlas (HPA; https://www.proteinatlas.org) to compare protein-level expression patterns 38.

### 2.3. Survival analysis using Cox proportional hazards model

To evaluate the independent prognostic impact of EZH2 expression in hepatocellular carcinoma (HCC), a multivariate Cox proportional hazards regression analysis was conducted. Covariates included in the model were age, sex, tumor grade, and EZH2 expression level. Patients with incomplete data for any of the variables were excluded from the analysis. Overall survival (OS) was defined as the interval from the date of diagnosis to the date of death or last follow-up. Hazard ratios (HRs) and 95% confidence intervals (CIs) were calculated, and statistical significance was defined as a two-sided p-value < 0.05.

### 2.4. TIMER database to estimate tumor-infiltrating immune cells

The TIMER database (Tumor Immune Estimation Resource; https://cistrome.shinyapps.io/timer/), which contains gene expression profiles from 10,897 samples across 32 cancer types obtained from The Cancer Genome Atlas (TCGA), was employed to estimate the infiltration levels of various immune cell types, including B cells, CD4⁺ T cells, CD8⁺ T cells, neutrophils, macrophages, and dendritic cells 39. In this study, the gene of the TIMER platform was utilized to analyze the correlation between EZH2 expression and the infiltration levels of these tumor-infiltrating immune cells in HCC samples.

### 2.5. DNA methylation of the EZH2 gene

DNA methylation data for the *EZH2* gene were retrieved from the UALCAN database and the UCSC Xena web platform (https://xenabrowser.net) 36, based on the Illumina Infinium HumanMethylation450 BeadChip platform. The methylation levels of individual CpG sites within the *EZH2* gene were analyzed according to tissue type (normal vs. tumor) and tumor grade (early vs. late stage). Methylation levels were represented as beta values ranging from 0 (completely unmethylated) to 1 (completely methylated). Samples were stratified into high methylation (beta value above the upper quartile) and low methylation (beta value below the upper quartile) groups. Kaplan-Meier survival analysis was conducted to evaluate the association between EZH2 methylation status and overall survival in patients with early- and late-stage hepatocellular carcinoma. All statistical analyses and data visualizations were performed using RStudio.

### *2.6.* Gene ontology (GO), Kyoto Encyclopedia of Genes and Genomes (KEGG) and Gene Set Enrichment Analysis (GSEA)

Computational analyses were conducted using GSEA software (version 4.3.2) and RStudio (version 4.4.1) to evaluate the statistical significance of biological signaling pathways. Gene Set Enrichment Analysis (GSEA) was performed to classify genes based on their correlation with the methylation status of cg08558971 and cg18416251 in HCC samples. HCC samples were further stratified into early- and late-stage groups based on histological tumor grade, where Grade 1 and Grade 2 were classified as early-stage, and Grade 3 and Grade 4 were classified as late-stage. This classification reflects the commonly observed progression in tumor differentiation, with higher grades generally indicating more advanced disease and poorer prognosis 40, 41. Each GSEA analysis was conducted with 1,000 permutations of gene sets to ensure robust enrichment statistics. Enriched pathways were ranked based on nominal p-values and normalized enrichment scores (NES), with a false discovery rate (FDR) < 0.05 considered statistically significant. To further investigate functional enrichment, Gene Ontology (GO) and Kyoto Encyclopedia of Genes and Genomes (KEGG) pathway analyses were performed using the clusterProfiler package in R. Two comparison groups were analyzed to identify shared signaling pathways associated with EZH2 methylation status, high methylation of cg08558971 in early-stage HCC combined with low methylation of cg18416251 in late-stage HCC, and low methylation of cg08558971 in early-stage HCC combined with high methylation of cg18416251 in late-stage HCC. Common genes involved in these pathways were identified to elucidate potential molecular mechanisms underlying EZH2-associated tumor progression.

### 2.7. Statistical analysis

All statistical analyses were performed using R software (version 4.4.1). The R packages utilized included *survminer*, *forestplot*, *pheatmap*, *ggplot2*, *clusterProfiler*, and *enrichplot*. A two-tailed p-value < 0.05 was considered statistically significant.

## 3. Results

### 3.1. Elevated *EZH2* expression is associated with unfavorable prognosis in HCC

**Figure 1:**
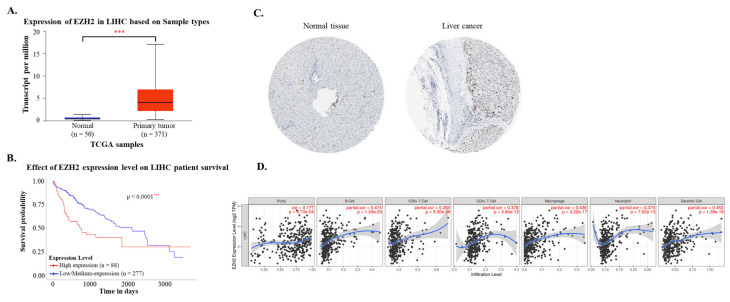
EZH2 mRNA and protein expression levels are elevated in tumor tissues and associated with poor survival and immune cell infiltration in hepatocellular carcinoma (HCC). (A) EZH2 mRNA expression levels are significantly higher in HCC tissues (n = 371) compared to normal liver tissues (n = 50), as analyzed using the UALCAN database (https://ualcan.path.uab.edu). (B) Kaplan-Meier survival analysis demonstrates poorer overall survival in HCC patients with high EZH2 expression (red) compared to those with low expression (blue). (C) Immunohistochemical staining shows elevated EZH2 protein expression in HCC tissues compared to normal liver tissues, based on data from The Human Protein Atlas (HPA; https://www.proteinatlas.org). (D) Correlation between EZH2 expression and the infiltration levels of immune cells in HCC was analyzed using the TIMER database, based on TCGA-LIHC data. Data are presented as mean ± SD. ***p < 0.001.

Using data from the TCGA-LIHC cohort accessed via the UALCAN platform, both mRNA and protein expression levels of EZH2 were found to be significantly elevated in hepatocellular carcinoma (HCC) tissues compared to normal liver tissues (Figure 1A and 1C). Immunohistochemical (IHC) analysis from the Human Protein Atlas further confirmed the increased EZH2 protein expression in tumor samples. Survival analysis indicated that high EZH2 expression was significantly associated with poorer overall prognosis in HCC patients (Figure 1B). In the multivariate Cox regression analysis, after adjusting for age, sex, tumor grade, and EZH2 expression levels, high EZH2 expression remained significantly associated with an increased risk of poor overall survival in HCC patients (Hazard ratio = 1.86, p = 0.0007) (Table 1). To further explore the potential involvement of EZH2 in tumor-immune interactions, we employed the TIMER database to assess correlations between EZH2 expression and immune cell infiltration in HCC. EZH2 expression was significantly and positively correlated with tumor purity (r = 0.177, p = 9.73 × 10⁻⁴), as well as with the infiltration levels of various immune cells, including B cells (r = 0.474, p = 1.24 × 10⁻²⁰), CD8⁺ T cells (r = 0.284, p = 9.3 × 10⁻⁸), CD4⁺ T cells (r = 0.378, p = 3.84 × 10⁻¹³), macrophages (r = 0.436, p = 3.22 × 10⁻¹⁷), neutrophils (r = 0.374, p = 7.02 × 10⁻¹³), and dendritic cells (DCs; r = 0.453, p = 1.38 × 10⁻¹⁸) (Figure 1D). These results suggest that EZH2 may contribute to HCC progression not only through its oncogenic overexpression but also via modulation of the tumor immune microenvironment.

### 3.2. Promoter methylation of EZH2 is associated with advanced-stage HCC

**Figure 2:**
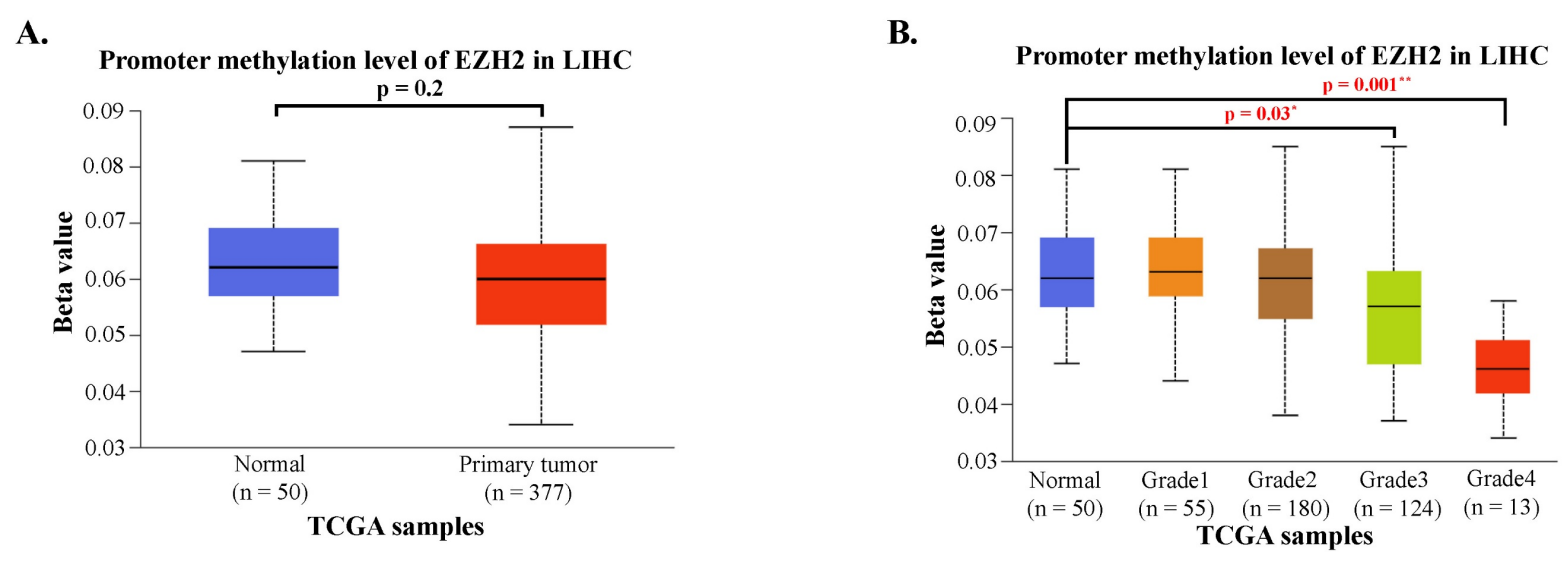
Promoter methylation levels of EZH2 are reduced in advanced tumor grades. (A) Promoter methylation levels of EZH2 show no significant differences between normal and tumor tissues when categorized by sample type. (B) Promoter methylation levels of EZH2 are significantly decreased in higher tumor grades (Grade 3 and Grade 4) compared to normal tissue. Data are presented as mean ± SD. **p < 0.01; *p < 0.05.

To investigate potential regulatory mechanisms of EZH2 expression, we analyzed the promoter methylation levels of EZH2 using the UALCAN platform. Although the methylation levels in the EZH2 promoter region were lower in HCC tissues compared to normal liver tissues, the difference was not statistically significant (Figure 2A). However, when HCC patients were stratified by tumor grade, a significant decrease in EZH2 promoter methylation was observed in advanced-stage tumors (Figure 2B). These findings suggest that promoter hypomethylation may contribute to the upregulation of EZH2 expression in advanced-stage HCC.

### 3.3. Inverse methylation patterns of *cg08558971* and *cg18416251* across HCC stages and association with patient prognosis

**Figure 3:**
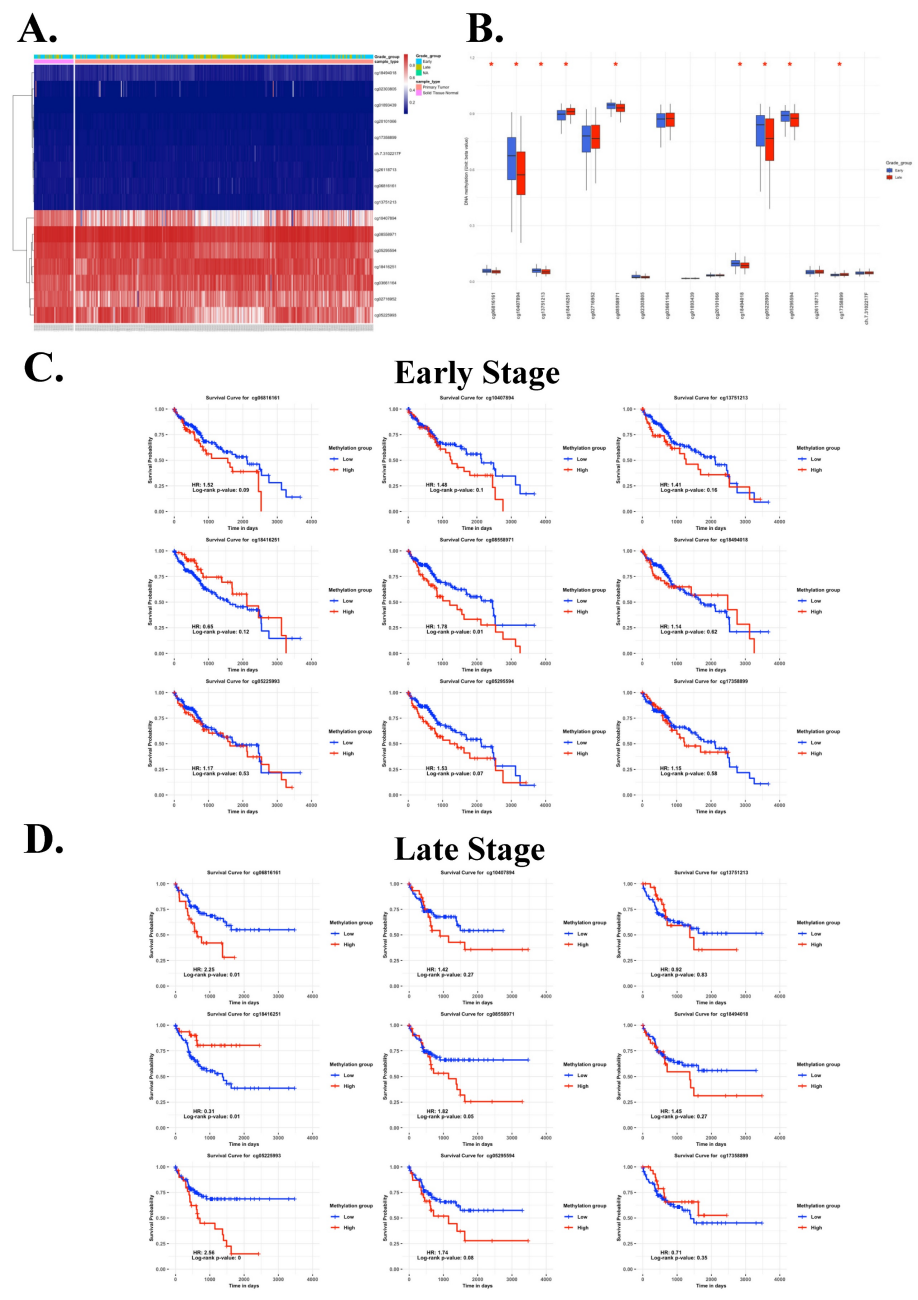
Inverse correlation of CpG sites cg08558971 and cg18416251 between early- and late-stage HCC and their prognostic significance. (A) Heatmap showing the promoter methylation levels of the EZH2 gene across HCC samples. (B) Differential methylation levels of individual CpG sites within the EZH2 promoter region. (C-D) Kaplan-Meier survival analysis illustrating the prognostic value of CpG sites cg08558971 and cg18416251 in HCC. Statistical significance was determined using the log-rank test, with p < 0.05 considered significant.

To further explore the epigenetic regulation of EZH2 in HCC, we analyzed individual CpG methylation sites across HCC samples. A heatmap was generated to visualize the clustering patterns and methylation levels of EZH2-associated CpG sites in tumor samples (Figure 3A). For stage classification, tumor grades 1 and 2 were defined as early-stage, while grades 3 and 4 were classified as late-stage. Bar plots demonstrated differential methylation levels of specific CpG sites between early- and late-stage tumors (Figure 3B). Survival analysis was conducted by stratifying patients into high and low methylation groups for each stage, revealing distinct associations between methylation levels and clinical outcomes (Figure 3C-D, Table 2). Notably, we observed an inverse correlation between methylation at cg08558971 in early-stage and cg18416251 in late-stage HCC. Collectively, these findings suggest that cg08558971 and cg18416251, two CpG sites located within the EZH2 gene, may have stage-specific epigenetic associations with EZH2 expression and contribute to the molecular landscape of HCC development and progression.

### 3.4. Consistent inverse methylation correlation of cg08558971 and cg18416251 in overall, early-, and late-stage HCC

To evaluate the methylation correlation between cg08558971 and cg18416251, we performed Pearson correlation analyses across overall, early-stage, and late-stage HCC samples. The results consistently demonstrated a statistically significant negative correlation between the two CpG sites. These findings suggest a potential inverse regulatory relationship between cg08558971 and cg18416251, implicating their involvement in critical signaling pathways associated with HCC progression.

### 3.5. Inverse enrichment of immune and metabolic pathways by methylation status of cg08558971 and cg18416251

To explore a potential inverse regulatory relationship between cg08558971 and cg18416251 in pathway involvement, we performed Gene Set Enrichment Analysis (GSEA) comparing early- and late-stage samples stratified by the methylation status of these two CpG sites. The results revealed distinct and opposing enrichment patterns, immune-related signaling pathways were enriched in samples with high methylation at cg08558971 and low methylation at cg18416251, whereas metabolic-related pathways were predominantly enriched in samples with low methylation at cg08558971 and high methylation at cg18416251. These findings suggest that cg08558971 and cg18416251 may play reciprocal roles in regulating immune and metabolic signaling during disease progression.

### 3.6. Shared genes reflect T cell differentiation and metabolic pathways across opposing methylation patterns

**Figure 6:**
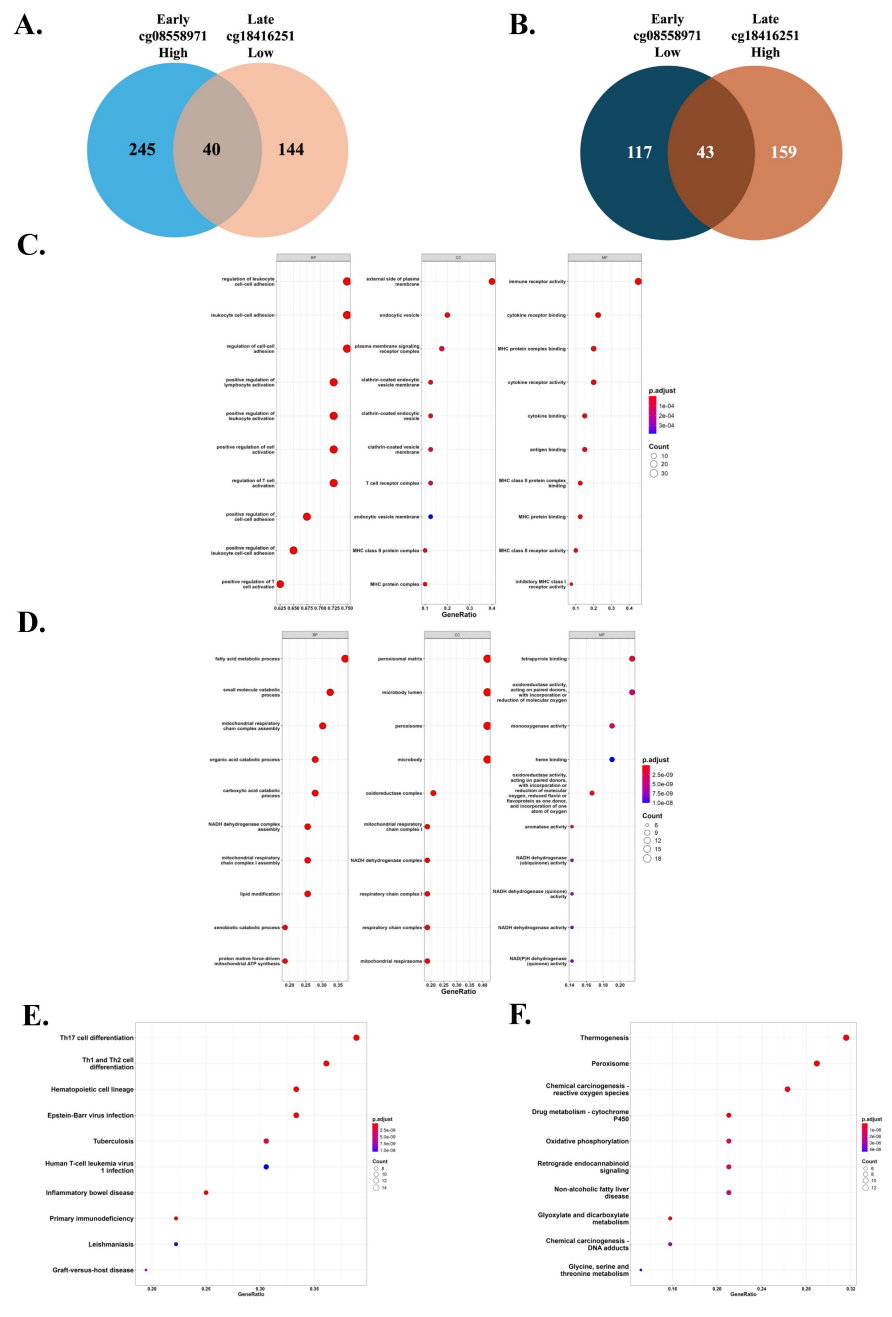
Common expression genes and enriched signaling pathways associated with differential methylation patterns of cg08558971 and cg18416251 across tumor stages. (A-B) Venn diagrams illustrating the overlap of common and differentially expressed genes (DEGs) between two methylation transitions that one is high methylation of cg08558971 in early-stage tumors and low methylation of cg18416251 in late-stage tumors (A), and the other is low methylation of cg08558971 in early-stage tumors and high methylation of cg18416251 in late-stage tumors (B). (C-D) Gene Ontology (GO) enrichment analysis of common expression genes identified in the transitions described in (A-B). Genes were significantly enriched in immune-related (C) and metabolism-related pathways (D). (E-F) Kyoto Encyclopedia of Genes and Genomes (KEGG) pathway enrichment analysis of common expression genes from both transitions. Genes also showed predominant enrichment in immune-related (E) and metabolism-related (F) signaling pathways.

We investigated genes commonly associated with two inverse DNA methylation patterns, one characterized by high methylation at cg08558971 and low methylation at cg18416251, and the other showing the opposite trend. A total of 40 genes were identified as commonly associated with the first pattern, while 43 genes were shared in the second pattern (Figure 6A-B). Subsequent Gene Ontology (GO) and Kyoto Encyclopedia of Genes and Genomes (KEGG) pathway analyses revealed that genes in the first pattern were enriched in immune-related pathways, particularly T helper cell differentiation pathways including Th1, Th2, and Th17 cell differentiation. In contrast, genes associated with the second pattern were predominantly enriched in metabolic pathways, such as thermogenesis and peroxisome function (Figure 6C-F). These findings suggest that the two opposing methylation patterns may influence tumor development through distinct biological processes, specifically immune regulation and metabolic modulation.

### 3.7. Association of EZH2 expression with enhanced T cell differentiation and suppressed metabolic pathways

**Figure 7:**
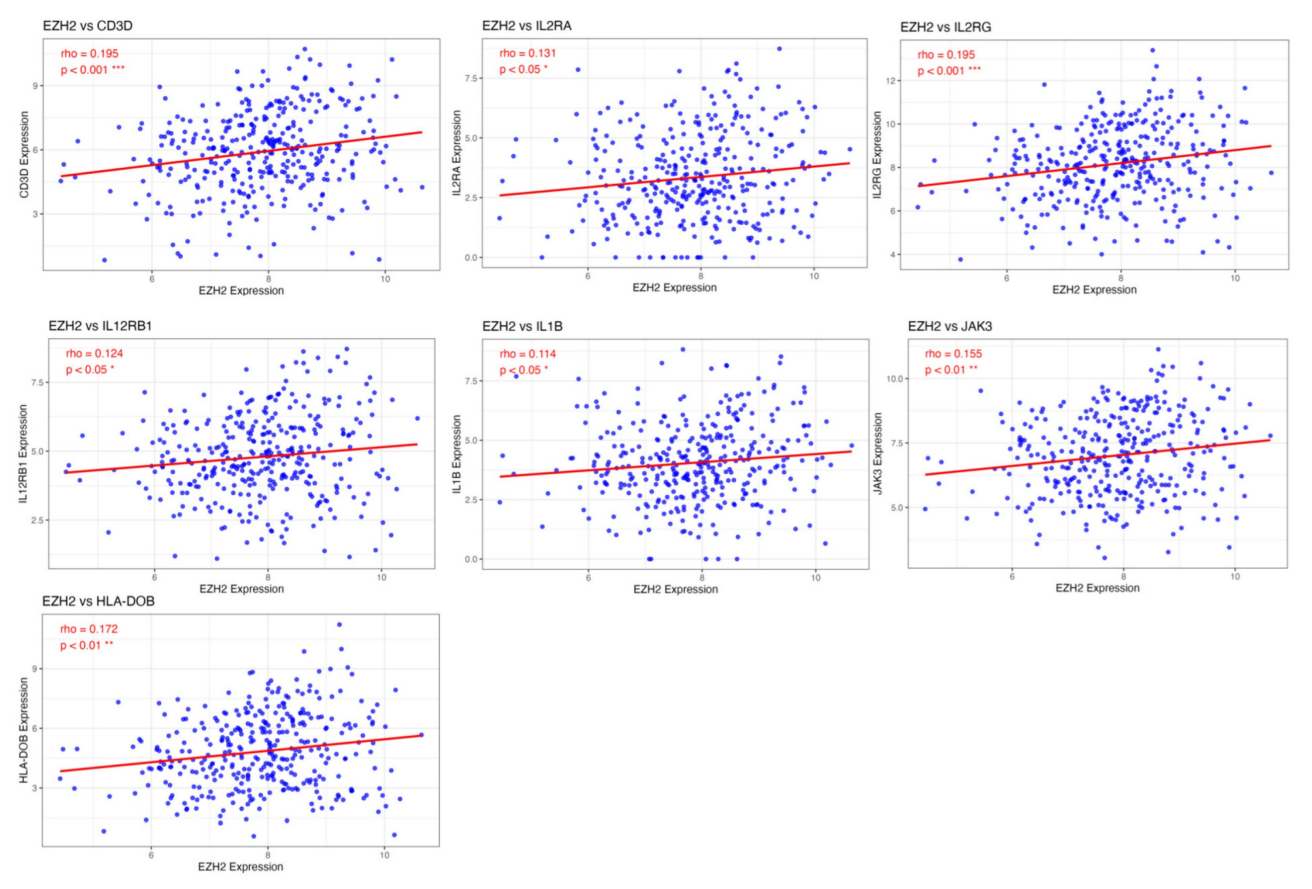
Immune-related genes positively correlated with EZH2 expression. Advanced analysis revealed that genes enriched in immune-related signaling pathways are positively correlated with EZH2 expression. These genes are primarily involved in T cell differentiation, particularly in Th17 cell differentiation. Statistical significance is indicated as follows, ***p < 0.001; **p < 0.01; *p < 0.05.

**Figure 8:**
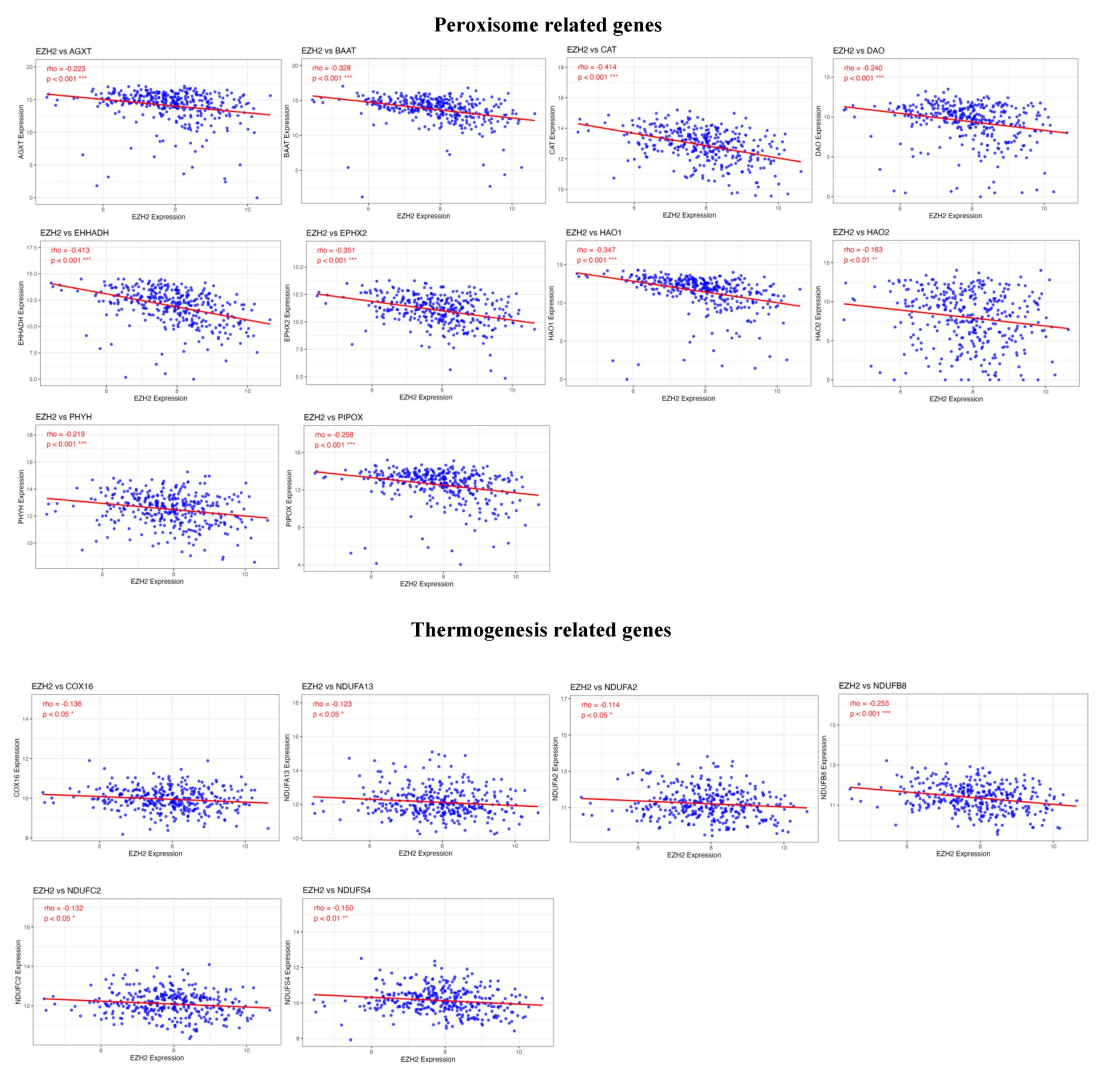
Metabolism-related genes negatively correlated with EZH2 expression. Advanced analysis demonstrated that genes enriched in metabolism-related signaling pathways are negatively correlated with EZH2 expression, especially those involved in peroxisome function and thermogenesis. Statistical significance is denoted as follows, ***p < 0.001; **p < 0.01; *p < 0.05.

To identify potential genes regulated by EZH2 that are involved in T cell differentiation and metabolic processes, we performed a correlation analysis between gene expression profiles and EZH2 expression. The results revealed a positive correlation between EZH2 expression and genes involved in T cell differentiation, particularly IL1B, a key gene associated with Th17 cell differentiation (Figure 7). In contrast, EZH2 expression was negatively correlated with genes related to peroxisome function and thermogenesis (Figure 8). These findings suggest that EZH2 may promote Th17 cell development and related immune responses, while simultaneously repressing metabolic processes such as peroxisome activity and thermogenesis, potentially influencing tumor progression.

## 4. Discussion

EZH2 is widely recognized as an oncogenic factor, characterized by its SET domain that facilitates trimethylation of histone H3 at lysine 27, a histone modification crucial for chromatin remodeling and gene silencing. Aberrant EZH2 expression has been associated with increased malignancy and poor prognosis in various cancers 42. Although the involvement of EZH2 in HCC has been previously reported, the underlying epigenetic regulatory mechanisms and key signaling pathways associated with its role in HCC progression remain incompletely understood. In our present study, we confirmed elevated EZH2 expression in HCC tissues, highlighting its significant association with adverse clinical outcomes. Further investigation into methylation patterns at specific CpG sites within the EZH2 promoter, cg08558971 and cg18416251, revealed distinct and inverse methylation profiles at different tumor stages, each significantly linked to overall survival. cg08558971 (promoter) and cg18416251 (intron 1) occupy distinct regulatory contexts. Promoter hypermethylation at cg08558971 could silence an upstream antisense element, indirectly fostering oncogenic signaling, whereas gene-body hypomethylation at cg18416251 is compatible with transcriptional up-regulation of EZH2 in advanced disease 43, 44. This stage-dependent switch reconciles the seemingly paradoxical association of both CpGs with poor survival. Specifically, hypermethylation at cg08558971 in early-stage HCC was associated with poorer prognosis, whereas hypomethylation at cg18416251 in late-stage tumors correlated with unfavorable outcomes, indicating stage-specific epigenetic regulatory mechanisms. GSEA revealed that these differential methylation states influenced immune-related pathways in one direction and metabolic pathways in the opposite. GO and KEGG analyses further identified that genes co-expressed with these methylation patterns were notably enriched in T-cell differentiation pathways, peroxisomal functions, and thermogenesis processes. Correlation analysis also showed that EZH2 expression was positively associated with genes related to Th17 cell differentiation and negatively associated with genes involved in peroxisomal activity and thermogenesis, suggesting that EZH2 may regulate the tumor microenvironment by modulating immune and metabolic pathways, thereby contributing to HCC progression.

Recent studies have suggested that EZH2 may promote tumor immune evasion by inducing the release of immunosuppressive cytokines and chemokines from tumor cells 45. Our analysis further proposes a possible connection between EZH2-related methylation patterns and T-cell differentiation, highlighting IL1B as a potential mediator. IL-1β is known to promote the differentiation and expansion of Th17 cells, which in turn can enhance tumor growth, angiogenesis, and metastasis 46-49. Th17 cells may display anti-tumour properties in selected settings 50, chronic hepatic inflammation skews them towards a pathogenic phenotype. High IL-17/IL-17RE levels independently predict early recurrence and poor survival in HCC 51. Consistently, EZH2-high tumours in our cohort exhibit an IL-1β/RORC-dominated Th17 signature that likely fosters tumour progression. Moreover, Th17 cells secrete cytokines such as IL-17A and IL-22, which contribute to tumor proliferation and immunosuppression 50. On the other hand, we also observed diminished activity of metabolic pathways, notably peroxisomal function and thermogenesis, in samples exhibiting higher EZH2 expression. Peroxisomes play pivotal roles in lipid metabolism and oxidative balance, and their dysfunction has been implicated in cancer development, possibly via mechanisms involving HIF-2α activation 52-56. Although peroxisomes have received less attention than mitochondria in cancer research, their role in liver cancer warrants further investigation. Thermogenesis, an energy-expending process primarily driven by beige and brown adipocytes, has been shown to alleviate hepatic inflammation and prevent the progression of non-alcoholic fatty liver disease (NAFLD) to HCC 57-59. Recent studies indicate that cold-induced thermogenesis may inhibit tumor growth by restricting glucose availability 60, suggesting that targeting thermogenic pathways could represent an innovative therapeutic approach. Our data show that high EZH2 is associated with suppression of peroxisome-related and thermogenic pathways, suggesting a shift from oxidative metabolism to glycolysis and lipid storage. This metabolic reprogramming can profoundly impact the tumor-immune microenvironment. Enhanced glycolysis in EZH2-high tumors not only accelerates lactate production and acidification of the TME but also results in competition with immune cells for nutrients (glucose, amino acids), effectively starving tumor-infiltrating lymphocytes 61. The lactate-rich, oxygen-poor milieu preferentially impairs effector T-cell function and proliferation, while favoring immunosuppressive cells such as M2-polarized macrophages and regulatory T cells. Concurrently, EZH2-mediated downregulation of peroxisomal β-oxidation may lead to accumulation of fatty acids and lipid droplets. Excess lipids in the TME can be taken up by dendritic cells and macrophages, driving them toward a lipid-laden, immunosuppressive state 62. Lipid-accumulated antigen-presenting cells have diminished capacity to prime anti-tumor T cells, and lipid-conditioned TAMs secrete factors (e.g., IL-6, TGF-β) that further dampen T-cell immunity. Moreover, the reduction in peroxisomal oxidative metabolism could exacerbate intracellular oxidative stress; high levels of ROS in the TME are known to inhibit T-cell responses and promote the expansion of suppressive myeloid cells 63. Consistent with these mechanisms, EZH2 has been implicated in multiple immune-evasive programs. Notably, EZH2 can epigenetically enforce the “Warburg” metabolic phenotype - for example, in glioma and liver cancer cells, EZH2 activation of HIF-1α and repression of mitochondrial regulators enhance glycolysis and lactate output 64. EZH2 also aids tumors in co-opting immune suppression via cytokine networks; a recent study in HCC demonstrated that EZH2, through cooperation with NF-κB, upregulates IL-6, driving MDSC accumulation and T-cell suppression 65. Taken together, these findings support a model in which EZH2-induced metabolic alterations, including heightened glycolysis, altered lipid metabolism, and impaired oxidative detoxification, collectively contribute to an immune privileged microenvironment that enables HCC to escape immune surveillance. However, the interaction between thermogenic adipocytes and HCC remains largely uncharacterized.

Despite these findings, our study has several limitations. First, the conclusions are based primarily on bioinformatic analyses, and the proposed mechanisms require further experimental validation. Second, it remains unclear whether the identified CpG sites are located within transcription factor binding regions of the EZH2 promoter or directly influence EZH2 expression. Lastly, the downstream genes and molecular mechanisms regulated by EZH2 have yet to be functionally verified. Future in vitro and in vivo studies are necessary to delineate the precise role of EZH2 in HCC development and to evaluate its potential as a therapeutic target. Collectively, our study demonstrates that EZH2 overexpression is associated with poor prognosis in HCC and is closely linked to immunometabolic regulation. We identified two CpG methylation sites with opposite patterns at different tumor stages, which may jointly contribute to HCC progression through modulation of T cell differentiation and metabolic reprogramming. These findings provide important insights into the epigenetic regulatory mechanisms of EZH2 and offer a foundation for future investigations into precision therapeutic strategies targeting the tumor microenvironment in HCC.

## 5. Conclusions

*EZH2* may serve as an independent prognostic biomarker in HCC, with its expression associated with inverse methylation patterns at specific CpG sites, cg08558971 and cg18416251 across different tumor stages. These methylation alterations may influence T cell differentiation and metabolic pathways, suggesting that EZH2 expression is regulated by epigenetic mechanisms during HCC progression and may play a critical role in tumor development.

## Figures and Tables

**Figure 4:**
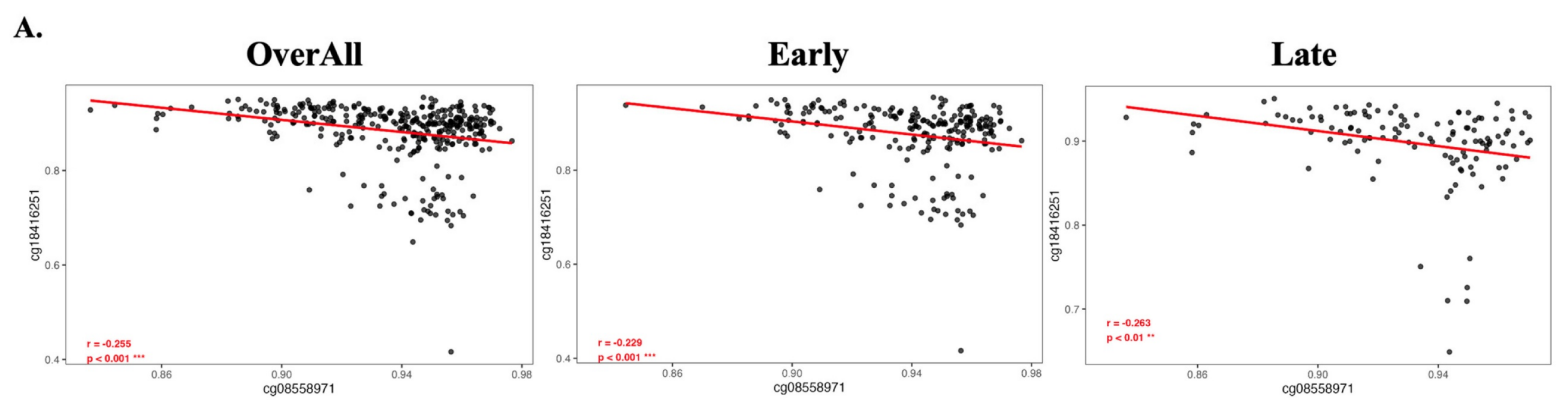
Negative correlation between cg08558971 and cg18416251 in HCC, consistent across overall, early-stage, and late-stage comparisons. Methylation levels of cg08558971 and cg18416251 exhibit a significant inverse correlation, regardless of disease stage. Data are presented as mean ± SD. ***p < 0.001; **p < 0.01.

**Figure 5:**
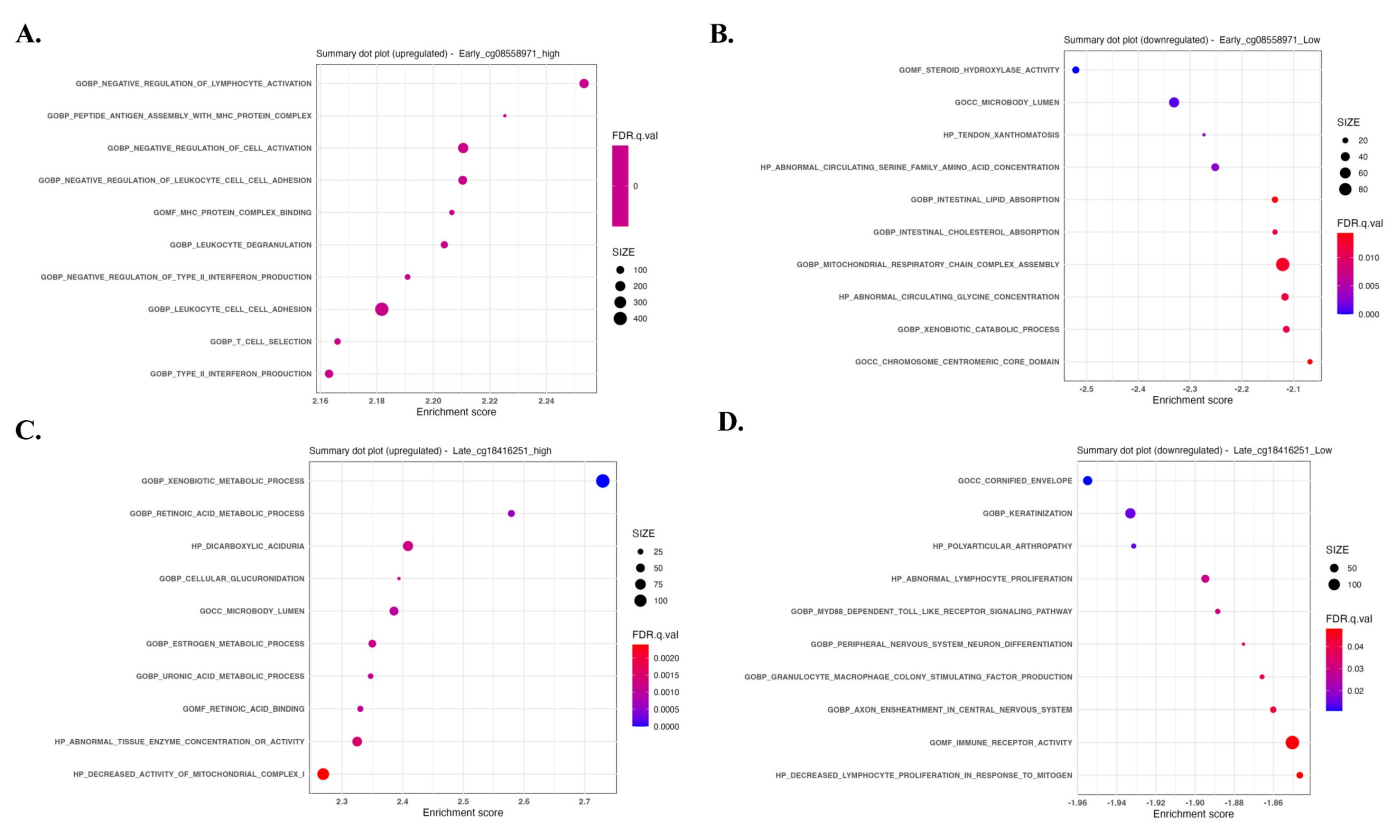
Gene sets and pathways enriched according to the methylation status of cg08558971 and cg18416251, revealing inverse associations with immune- and metabolism-related signaling. Gene Set Enrichment Analysis (GSEA) identified significantly enriched signaling pathways associated with differential methylation of cg08558971 and cg18416251. (A-B) Enriched pathways in high and low methylation groups of cg08558971. (C-D) Enriched pathways in high and low methylation groups of cg18416251. Significantly enriched pathways were determined using a nominal p-value < 0.05.

**Table 1:**
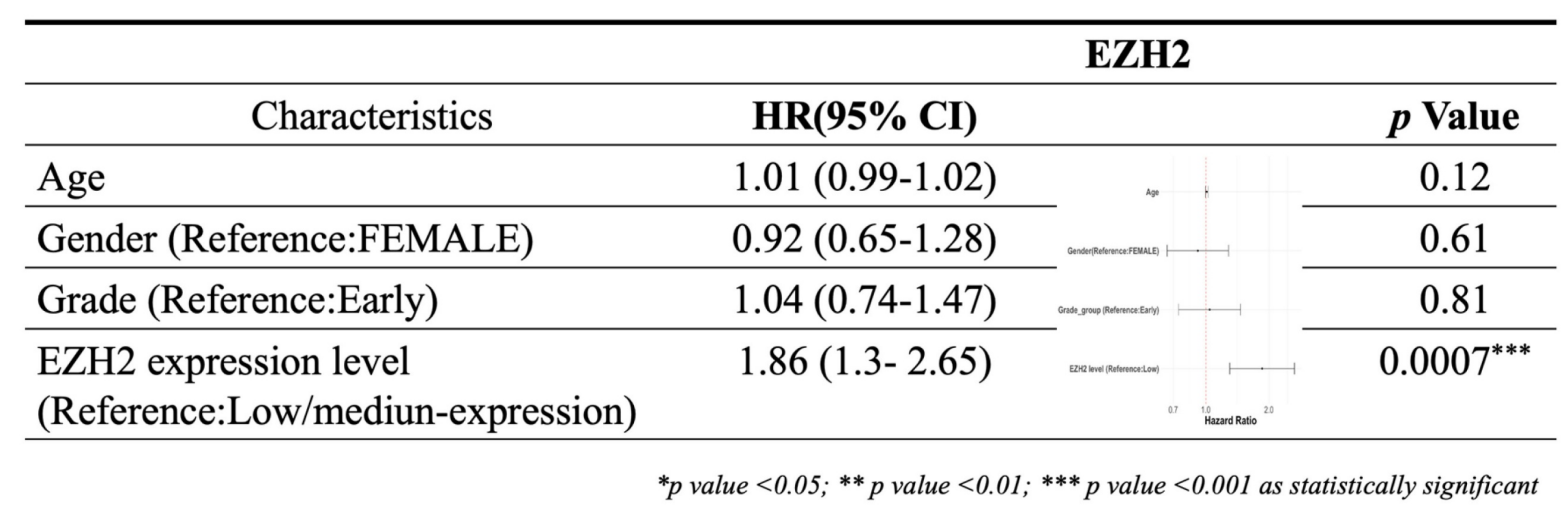
Multivariate Cox regression analysis of clinicopathological features with OS in the TCGA datasets.

**Table 2:**
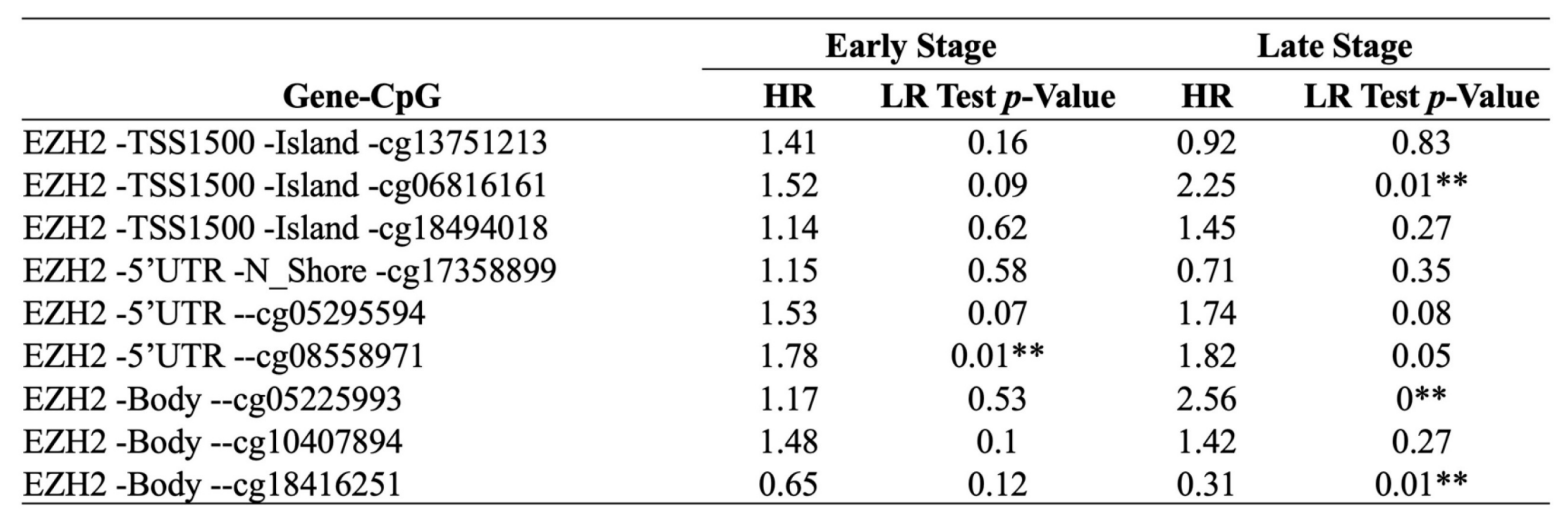
Prognostic of single CpG sites in the EZH2 gene family across different stages of LIHC using the HumanMethylation450 platform

## References

[1] Vogel A, Meyer T, Sapisochin G, Salem R, Saborowski A (2022). Hepatocellular carcinoma. Lancet.

[2] Lee HL, Chien YC, Wang HL, Hua CH, Liu LC, Wu GW (2022). Analysis of MUC6 Genetic Variants on the Clinicopathologic Characteristics of Patients with Hepatocellular Carcinoma. J Cancer.

[3] Siegel RL, Kratzer TB, Giaquinto AN, Sung H, Jemal A (2025). Cancer statistics, 2025. CA Cancer J Clin.

[4] Anstee QM, Reeves HL, Kotsiliti E, Govaere O, Heikenwalder M (2019). From NASH to HCC: current concepts and future challenges. Nat Rev Gastroenterol Hepatol.

[5] Bayram AA, Al-Dahmoshi HOM, Al-Khafaji NSK, Al Mammori RTO, Al-Shimmery AHS, Saki M (2021). Study of the D-dimer, C-reactive protein, and autoantibodies markers among HBV infected patients in Babylon province, Iraq. Biomedicine (Taipei).

[6] Ling Y, Zhu J, Gao L, Liu Y, Zhu C, Li R (2013). The silence of MUC2 mRNA induced by promoter hypermethylation associated with HBV in Hepatocellular Carcinoma. BMC Med Genet.

[7] Llovet JM, Kelley RK, Villanueva A, Singal AG, Pikarsky E, Roayaie S (2021). Hepatocellular carcinoma. Nat Rev Dis Primers.

[8] Zhang WZ, Chin KY, Zakaria R, Hassan NH (2025). Strategies for Pain Management in Hepatocellular Carcinoma Patients Undergoing Transarterial Chemoembolisation: A Scoping Review of Current Evidence. Healthcare (Basel).

[9] Zheng J, Wang S, Xia L, Sun Z, Chan KM, Bernards R (2025). Hepatocellular carcinoma: signaling pathways and therapeutic advances. Signal Transduct Target Ther.

[10] Chi H, Zhao S, Yang J, Gao X, Peng G, Zhang J (2023). T-cell exhaustion signatures characterize the immune landscape and predict HCC prognosis via integrating single-cell RNA-seq and bulk RNA-sequencing. Front Immunol.

[11] Gao B, Lu Y, Lai X, Xu X, Gou S, Yang Z (2025). Metabolic reprogramming in hepatocellular carcinoma: mechanisms of immune evasion and therapeutic implications. Front Immunol.

[12] Huang Y, Ge W, Zhou J, Gao B, Qian X, Wang W (2021). The Role of Tumor Associated Macrophages in Hepatocellular Carcinoma. J Cancer.

[13] Xia Z, Chen S, He M, Li B, Deng Y, Yi L (2023). Editorial: Targeting metabolism to activate T cells and enhance the efficacy of checkpoint blockade immunotherapy in solid tumors. Front Immunol.

[14] Zhang X, Zhuge J, Liu J, Xia Z, Wang H, Gao Q (2023). Prognostic signatures of sphingolipids: Understanding the immune landscape and predictive role in immunotherapy response and outcomes of hepatocellular carcinoma. Front Immunol.

[15] Heyn H, Esteller M (2013). EZH2: an epigenetic gatekeeper promoting lymphomagenesis. Cancer Cell.

[16] Yu YL, Su KJ, Hsieh YH, Lee HL, Chen TY, Hsiao PC (2013). Effects of EZH2 polymorphisms on susceptibility to and pathological development of hepatocellular carcinoma. PLoS One.

[17] Bai J, Chen J, Ma M, Cai M, Xu F, Wang G (2014). Inhibiting enhancer of zeste homolog 2 promotes cellular senescence in gastric cancer cells SGC-7901 by activation of p21 and p16. DNA Cell Biol.

[18] Cao Q, Yu J, Dhanasekaran SM, Kim JH, Mani RS, Tomlins SA (2008). Repression of E-cadherin by the polycomb group protein EZH2 in cancer. Oncogene.

[19] Kim KH, Roberts CW (2016). Targeting EZH2 in cancer. Nat Med.

[20] Sauvageau M, Sauvageau G (2010). Polycomb group proteins: multi-faceted regulators of somatic stem cells and cancer. Cell Stem Cell.

[21] Fujii S, Ito K, Ito Y, Ochiai A (2008). Enhancer of zeste homologue 2 (EZH2) down-regulates RUNX3 by increasing histone H3 methylation. J Biol Chem.

[22] Hu Y, Dong Z, Liu K (2024). Unraveling the complexity of STAT3 in cancer: molecular understanding and drug discovery. J Exp Clin Cancer Res.

[23] Kim J, Lee Y, Lu X, Song B, Fong KW, Cao Q (2018). Polycomb- and Methylation-Independent Roles of EZH2 as a Transcription Activator. Cell Rep.

[24] Lee ST, Li Z, Wu Z, Aau M, Guan P, Karuturi RK (2011). Context-specific regulation of NF-kappaB target gene expression by EZH2 in breast cancers. Mol Cell.

[25] Moretti RM, Montagnani Marelli M, Motta M, Limonta P (2002). Role of the orphan nuclear receptor ROR alpha in the control of the metastatic behavior of androgen-independent prostate cancer cells. Oncol Rep.

[26] Pan YM, Wang CG, Zhu M, Xing R, Cui JT, Li WM (2016). STAT3 signaling drives EZH2 transcriptional activation and mediates poor prognosis in gastric cancer. Mol Cancer.

[27] Ren G, Baritaki S, Marathe H, Feng J, Park S, Beach S (2012). Polycomb protein EZH2 regulates tumor invasion via the transcriptional repression of the metastasis suppressor RKIP in breast and prostate cancer. Cancer Res.

[28] Savage AK, Constantinides MG, Han J, Picard D, Martin E, Li B (2008). The transcription factor PLZF directs the effector program of the NKT cell lineage. Immunity.

[29] Taniguchi H, Jacinto FV, Villanueva A, Fernandez AF, Yamamoto H, Carmona FJ (2012). Silencing of Kruppel-like factor 2 by the histone methyltransferase EZH2 in human cancer. Oncogene.

[30] Xu K, Wu ZJ, Groner AC, He HH, Cai C, Lis RT (2012). EZH2 oncogenic activity in castration-resistant prostate cancer cells is Polycomb-independent. Science.

[31] Yan J, Ng SB, Tay JL, Lin B, Koh TL, Tan J (2013). EZH2 overexpression in natural killer/T-cell lymphoma confers growth advantage independently of histone methyltransferase activity. Blood.

[32] Mortezaee K (2025). EZH2 regulatory roles in cancer immunity and immunotherapy. Pathol Res Pract.

[33] Nutt SL, Keenan C, Chopin M, Allan RS (2020). EZH2 function in immune cell development. Biol Chem.

[34] Erickson BJ (2016). The Cancer Genome Atlas Liver Hepatocellular Carcinoma Collection (TCGA-LIHC) (Version 5) [Data set]. The Cancer Imaging Archive.

[35] Chandrashekar DS, Karthikeyan SK, Korla PK, Patel H, Shovon AR, Athar M (2022). UALCAN: An update to the integrated cancer data analysis platform. Neoplasia.

[36] Goldman MJ, Craft B, Hastie M, Repecka K, McDade F, Kamath A (2020). Visualizing and interpreting cancer genomics data via the Xena platform. Nat Biotechnol.

[37] Chandrashekar DS, Bashel B, Balasubramanya SAH, Creighton CJ, Ponce-Rodriguez I, Chakravarthi B (2017). UALCAN: A Portal for Facilitating Tumor Subgroup Gene Expression and Survival Analyses. Neoplasia.

[38] Uhlen M, Oksvold P, Fagerberg L, Lundberg E, Jonasson K, Forsberg M (2010). Towards a knowledge-based Human Protein Atlas. Nat Biotechnol.

[39] Li T, Fan J, Wang B, Traugh N, Chen Q, Liu JS (2017). TIMER: A Web Server for Comprehensive Analysis of Tumor-Infiltrating Immune Cells. Cancer Res.

[40] Matsuo K, Mandelbaum RS, Machida H, Purushotham S, Grubbs BH, Roman LD (2018). Association of tumor differentiation grade and survival of women with squamous cell carcinoma of the uterine cervix. J Gynecol Oncol.

[41] Zhang GX, Ding XS, Wang YL (2023). Prognostic model of hepatocellular carcinoma based on cancer grade. World J Clin Cases.

[42] Liu Y, Yang Q (2023). The roles of EZH2 in cancer and its inhibitors. Med Oncol.

[43] Jones PA (2012). Functions of DNA methylation: islands, start sites, gene bodies and beyond. Nat Rev Genet.

[44] Wu SY, Xie ZY, Yan LY, Liu XF, Zhang Y, Wang DA (2022). The correlation of EZH2 expression with the progression and prognosis of hepatocellular carcinoma. BMC Immunol.

[45] Kang N, Eccleston M, Clermont PL, Latarani M, Male DK, Wang Y (2020). EZH2 inhibition: a promising strategy to prevent cancer immune editing. Epigenomics.

[46] Chung Y, Chang SH, Martinez GJ, Yang XO, Nurieva R, Kang HS (2009). Critical regulation of early Th17 cell differentiation by interleukin-1 signaling. Immunity.

[47] Miyahara Y, Odunsi K, Chen W, Peng G, Matsuzaki J, Wang RF (2008). Generation and regulation of human CD4+ IL-17-producing T cells in ovarian cancer. Proc Natl Acad Sci U S A.

[48] Rebe C, Ghiringhelli F (2020). Interleukin-1beta and Cancer. Cancers (Basel).

[49] Dmitrieva-Posocco O, Dzutsev A, Posocco DF, Hou V, Yuan W, Thovarai V (2019). Cell-Type-Specific Responses to Interleukin-1 Control Microbial Invasion and Tumor-Elicited Inflammation in Colorectal Cancer. Immunity.

[50] Anvar MT, Rashidan K, Arsam N, Rasouli-Saravani A, Yadegari H, Ahmadi A (2024). Th17 cell function in cancers: immunosuppressive agents or anti-tumor allies?. Cancer Cell Int.

[51] Liao R, Sun J, Wu H, Yi Y, Wang JX, He HW (2013). High expression of IL-17 and IL-17RE associate with poor prognosis of hepatocellular carcinoma. J Exp Clin Cancer Res.

[52] Park H, He A, Tan M, Johnson JM, Dean JM, Pietka TA (2019). Peroxisome-derived lipids regulate adipose thermogenesis by mediating cold-induced mitochondrial fission. J Clin Invest.

[53] Lauer C, Volkl A, Riedl S, Fahimi HD, Beier K (1999). Impairment of peroxisomal biogenesis in human colon carcinoma. Carcinogenesis.

[54] Keller J-M, Cablé S, El Bouhtoury F, Heusser S, Scotto C, Armbruster L (1993). Peroxisome through cell differentiation and neoplasia. Biology of the Cell.

[55] Litwin JA, Beier K, Volkl A, Hofmann WJ, Fahimi HD (1999). Immunocytochemical investigation of catalase and peroxisomal lipid beta-oxidation enzymes in human hepatocellular tumors and liver cirrhosis. Virchows Arch.

[56] Walter KM, Schonenberger MJ, Trotzmuller M, Horn M, Elsasser HP, Moser AB (2014). Hif-2alpha promotes degradation of mammalian peroxisomes by selective autophagy. Cell Metab.

[57] Zhang X, Hu Y (2023). Mitochondrial thermogenesis in cancer cells. Oncologie.

[58] Yin X, Chen Y, Ruze R, Xu R, Song J, Wang C (2022). The evolving view of thermogenic fat and its implications in cancer and metabolic diseases. Signal Transduct Target Ther.

[59] Ye J, Wu S, Pan S, Huang J, Ge L (2020). Risk scoring based on expression of long non-coding RNAs can effectively predict survival in hepatocellular carcinoma patients with or without fibrosis. Oncol Rep.

[60] Chen Z, Kang Y (2023). Cold snap for cancer: cold-induced brown fat thermogenesis starves tumor growth. Signal Transduct Target Ther.

[61] Caini P, Carloni V (2025). Metabolism and Immune Suppressive Response in Liver Cancer. Biomedicines.

[62] Luo Q, Zheng N, Jiang L, Wang T, Zhang P, Liu Y (2020). Lipid accumulation in macrophages confers protumorigenic polarization and immunity in gastric cancer. Cancer Sci.

[63] Malla R, Kumari S, Ganji SP, Srilatha M, Nellipudi HR, Nagaraju GP (2024). Reactive oxygen species of tumor microenvironment: Harnessing for immunogenic cell death. Biochim Biophys Acta Rev Cancer.

[64] Zhang T, Gong Y, Meng H, Li C, Xue L (2020). Symphony of epigenetic and metabolic regulation-interaction between the histone methyltransferase EZH2 and metabolism of tumor. Clin Epigenetics.

[65] Muliawan GK, Lee TK (2024). The roles of cancer stem cell-derived secretory factors in shaping the immunosuppressive tumor microenvironment in hepatocellular carcinoma. Front Immunol.

